# Transcriptional analysis of CRISPR I-B arrays of *Leptospira interrogans* serovar Lai and its processing by Cas6

**DOI:** 10.3389/fmicb.2022.960559

**Published:** 2022-07-29

**Authors:** Aman Prakash, Manish Kumar

**Affiliations:** Department of Biosciences and Bioengineering, Indian Institute of Technology Guwahati, Guwahati, India

**Keywords:** *Leptospira*, CRISPR I-B, repeats, transcription, pre-crRNA, Cas6 endoribonuclease, crRNA biogenesis, mature crRNA

## Abstract

In the genome of various *Leptospira interrogans serovars*, the subtype I-B locus of CRISPR-Cas possesses either one or multiple CRISPR arrays. *In silico* database (CRISPRCasdb) for predicting CRISPR-Cas reveals seven CRISPR arrays in *L. interrogans* serovar Lai positioned between the two independent *cas*-operons. Here, we present the redefined repeat-spacer boundaries of the CRISPR subtype I-B locus of serovar Lai. Such refinement of boundaries of arrays in serovar Lai was done after comparison with the characterized array of another serovar Copenhageni and the manual analysis of CRISPR flanking sequences. Using the reverse transcription-PCR (RT-PCR), we account that the seven CRISPR are transcriptionally active in serovar Lai. Our RT-PCR and quantitative real-time PCR analysis of transcripts in serovar Lai indicated that seven CRISPR of subtype I-B transcribe together as a single precursor unit. Moreover, the cleavage of the two miniature pre-crRNA of the subtype I-B by Cas6 demonstrates the biogenesis of the expected size of mature crRNA essential for the guided interference of foreign DNA. This study features insight into transcription direction and the crRNA biogenesis in serovar Lai essential for RNA-mediated interference of invading nucleic acids.

## Introduction

CRISPR-Cas acquires the RNA-based adaptive immunity in prokaryotes against mobile foreign genetic material (Pourcel et al., 2005). A set of *cas* genes, a leader sequence, and the CRISPR array are the functional elements of the CRISPR-Cas immune system. A CRISPR comprises an array of direct repeats segregated by distinct spacer sequences, with a preceding leader sequence (Pourcel et al., 2005; Garneau et al., 2010; Yosef et al., 2012; Makarova et al., 2015). CRISPR immunity is led through three molecular stages: adaptation (or acquisition), expression (or CRISPR RNA biogenesis), and interference. An explicit region of foreign nucleic acid (protospacer) is embodied as a memory card at the leader’s proximal end during the adaptation stage (Yosef et al., 2012; Wang et al., 2015; Hille et al., 2018). In the expression stage, the CRISPR array is transcribed as a precursor CRISPR RNA (pre-crRNA) molecule and processed into mature crRNAs by the Cas endoribonuclease (Charpentier et al., 2015; Hochstrasser and Doudna, 2015). After that, the crRNA forms a complex with the Cas effector proteins in the final stage (interference) to interfere with the cognate alien nucleic acids (Barrangou et al., 2007; Hale et al., 2009; Garneau et al., 2010; Hille et al., 2018). The CRISPR-Cas require a protospacer adjacent motif (PAM) to recognize, differentiate, and eliminate specific foreign DNA (Westra et al., 2013). Although the length of PAMs can vary from two to six nucleotides, the most commonly reported PAM size is three to four nucleotides (Nishimasu et al., 2017). With the identification of a rising number of *cas* genes, the CRISPR-Cas systems have been divided into two classes (Class 1 and Class 2), six types (Type I-VI), and 33 subtype variants based on the different arrangements of *cas* genes and effector complex subunits (Makarova et al., 2020).

Several studies have shown the involvement of the CRISPR-cas system in regulating bacterial pathogenesis (Sampson et al., 2013; Sampson and Weiss, 2014). In a comparative genomics study among the saprophytic and infectious groups of *Leptospira*, CRISPR-Cas systems were identified mainly in the pathogenic *Leptospira* species (Fouts et al., 2016). The pathogenic species of the genus *Leptospira* causes leptospirosis, a zoonotic disease of global importance (Da and Levett, 2015). The presence of the CRISPR-Cas systems has been inferred as the virulence factor in pathogenic *Leptospira* (Fouts et al., 2016). Understanding *Leptospira* pathogenesis is still confined due to the lack of efficient genetic manipulation tools (Fernandes et al., 2021). With the advent of a shuttle vector (pMaOri), a new strategy to genetically manipulate *Leptospira* has been developed where episomal delivery of CRISPR-Cas9 (type II) was possible (Pappas et al., 2015). However, the type II application is limited to very few bacteria because the Cas9 induces double-strand breaks (DSBs) at the target DNA of the host, which must be repaired for cell viability (Lieber, 2010). In the *Leptospira* genome, owing to the absence of a non-homologous end-joining repair (NHEJ) system for DSBs, Cas9-induced DSBs were found to be lethal (Fernandes et al., 2019). To withstand the lethality, an inactive variant (dead) of CRISPR-dCas9 (CRISPRi) was employed for the targeted genetic manipulation in *Leptospira* spp. (Shapiro et al., 2018; Fernandes et al., 2021). However, the CRISPRi technology is limited to gene silencing (Zhao et al., 2017). Recently, CRISPR-Cas9 DSB lethality in *Leptospira* has been surpassed by the concomitant expression of the *Mycobacterium* NHEJ repair system (Fernandes and Nascimento, 2022). However, the tedious conjugation process and fastidious growth of *Leptospira* often lead to low efficiency of genetic manipulation (Fernandes et al., 2021).

Harnessing endogenous CRISPR-Cas types I and III systems of prokaryotes for genome editing is an attractive strategy to overcome the limitations of gene editing by type II Cas9 technology (Li et al., 2016). The pre-requisite of heterologous expression of Cas proteins (potentially toxic) can be surpassed inside the prokaryotes while exploiting the endogenous CRISPR-based method (Maikova et al., 2019). To date, an endogenous CRISPR-Cas system (subtypes I-A, I-B, or III-B) has been successfully applied for genome editing in several archaea and *Clostridium* spp. (Li et al., 2016; Pyne et al., 2016; Cheng et al., 2017; Maikova et al., 2019). Nevertheless, reprogramming the endogenous CRISPR-Cas system for genome editing relies on understanding the RNA-mediated immunity process. Thus, the molecular details of CRISPR arrays such as CRISPR transcription and orientation, repeat-spacer boundaries, and the PAM sequence are prerequisites to repurpose the endogenous CRISPR-Cas systems in *Leptospira* for genome editing. Such knowledge regarding the CRISPR-Cas system of pathogenic *Leptospira* will be advantageous in developing a genetic tool to understand gene function and pathogenesis at a higher efficiency. In the genus *Leptospira* and the genome of its infectious serovars (svs.), three variants of CRISPR/Cas type I systems (subtypes I-B, –C, and –E) are prevalent (Makarova et al., 2011; Xiao et al., 2019). In addition, recently CRISPR-Cas type V was recorded in a saprophytic *Leptospira* strain (*L. biflexa*) (Martínez Arbas et al., 2021). Serovars of *L. interrogans* harbor two subtypes (I-B and I-C) of the type I system; however, CRISPR arrays were identified only at the I-B locus (Xiao et al., 2019). Thus, it was speculated that the CRISPR-Cas I-B in *L. interrogans* might be sufficient for CRISPR immunity. In contrast, type I-C possibly carries out other unknown functions in *L. interrogans* (Xiao et al., 2019). In an *in silico* study of 41 *Leptospira* strains, 42% (48 out of 114) of the total identified CRISPR arrays were found more than 10 kb away from any *cas* gene (Xiao et al., 2019). Such isolated CRISPR arrays were referred to as orphan arrays (Zhang and Ye, 2017). In the genome of the pathogenic *L. interrogans* svs., such as Copenhageni and Lai, the two *cas* operons of the subtype I-B locus span a hypervariable region that contains either one or multiple CRISPR (Xiao et al., 2019). Previous studies from our group have defined the architecture CRISPR-Cas I-B locus in *L. interrogans* sv. Copenhageni strain Fiocruz L1-130 that comprises eight *cas* genes (*cas1*-*cas8*) and a single CRISPR (LIC_Cr^2^) (Dixit et al., 2016). The orientation of LIC_Cr^2^ pre-crRNA was along with the *cas* operons (Prakash and Kumar, 2021). Among the Cas proteins (LinCas1-LinCas8) associated with the I-B system of sv. Copenhageni, LinCas1 (Dixit et al., 2021b), LinCas2 (Dixit et al., 2016), LinCas4 (Dixit et al., 2021a), LinCas6 (Prakash and Kumar, 2021), and LinCas7 (Hussain and Kumar, 2022) have been characterized to date. In this study, in the genome of *L. interrogans* sv. Lai, the possible CRISPRs were predicted using the CRISPRCasdb database and was compared with the previously characterized I-B array of sv. Copenhageni. After that, the transcription of CRISPR I-B arrays in sv. Lai was analyzed using the reverse transcription-polymerase chain reaction (RT-PCR) technique. In addition, the processing of miniature pre-crRNA of sv. Lai has been studied using Cas6 to generate mature crRNAs.

## Materials and methods

### Bacterial strains and nucleic acid isolation

*Leptospira* strains (*L. interrogans* sv. Copenhageni strain Fiocruz L1-130 and *L. interrogans* sv. Lai strain 56601) were grown, maintained, and subcultured in the laboratory as described previously (Ghosh et al., 2018a,b). The cultures of leptospires strains were used in genomic DNA or total RNA isolation. The *Escherichia coli* strain (DH5α) was used for cloning and transformation.

### Bioinformatics analysis

From the available genomic sequence of sv. Copenhageni and Lai, the CRISPRCasdb (Pourcel et al., 2020) defined CRISPR repeats and spacer sequences were extracted. Multiple sequence alignments (MSA) of repeats and spacers’ nucleotide sequences were performed using Clustal Omega (Madeira et al., 2019), and the graphic images of aligned sequences were obtained using the ESPript program (version 3.0) (Robert and Gouet, 2014). The WebLogo tool (Crooks et al., 2004) was used to construct a logo of eight nucleotides flanking (5′ and 3′) protospacers that were retrieved via the CRISPRTarget (Biswas et al., 2013) tool. The primers were designed manually or using the “OligoPerfect Primer Designer” tool of Thermo Fisher Scientific.

### Reverse transcription-PCR (reverse transcription-polymerase chain reaction) and quantitative real-time PCR

The complementary DNA (cDNA) was synthesized from the total RNA (1 μg) of *Leptospira* (sv. Copenhageni or Lai) using the random hexamers or spacer-specific primer in a reverse transcription reaction as described previously (Ghosh et al., 2018a; Prakash and Kumar, 2021). Under similar conditions, an additional reaction was set up without the reverse transcriptase. Additional reaction served as a control (negative) in RT-PCR or qPCR to rule out the possibility of gDNA contamination in the purified RNA transcripts. The diluted (5-folds) and undiluted cDNA obtained were used as a template to perform qPCR and RT-PCR, respectively. Products of RT-PCR experiments were resolved onto 2% agarose gel. The qPCR analysis of CRISPR transcripts was performed according to established laboratory protocol (Ghosh et al., 2018a), where the transcripts of the target CRISPR were normalized with the *16S rRNA* (*rrs1*) of *Leptospira* using the 2^–ΔΔ*CT*^ method (Livak and Schmittgen, 2001). The CRISPR transcripts were calculated per 10^6^ copies of the *16S rRNA* of respective *Leptospira* svs. For statistical analysis, two independent experiments were performed in quadruplets.

### *In vitro* synthesis of precursor CRISPR RNAs

Two miniature CRISPR DNA corresponding to LA_Cr^6^ R2R4 (178 bp) and LA_Cr^12^ R2R3 (107 bp) were amplified through nested PCR using primer sets described in Table 1. The DNA fragments were cloned in a transcription vector pTZ57R/T between *Hin*dIII and *Kpn*I restriction sites, and the generated plasmid constructs were outsourced for sequencing. Pre-crRNAs were synthesized *in vitro* after linearizing each plasmid construct with *Kpn*I, as described previously (Prakash and Kumar, 2021). The two miniature pre-crRNA transcript [LA_Cr^6^ R2R4 (188 nt) and LA_Cr^12^ R2R3 (117 nt)] at its 5′ end also contains a vector-derived 10 nt sequence (5′GGGAAAGCUU3′).

**TABLE 1:** Oligos used in this study.

Primer names	Sequences (5′–3′)	Purpose
LA_Cr^6^ S1 forward (^6^S1_*f*_)	CCGTTCTGATTTTTTCTTTTCCT	Detection of CRISPR I-B of serovar Lai through PCR or RT-PCR
LA_Cr^6^ S3 reverse (^6^S3_*r*_)	GCGAGCATCGGTAGTTTTACC	
LA_Cr^7^ S1 forward (^7^S1_*f*_)	TTGATTGGTGCAGTTGTGCTT	
LA_Cr^7^ S4 reverse (^7^S4_*r*_)	TACGCCGGTTCCTCTTTTTTG	
LA_Cr^9^ S1 forward (^9^S1_*f*_)	AAAGACAAATCGGTTCATTTGA	
LA_Cr^9^ S4 reverse (^9^S4_*r*_)	TTTACGTTTTGAGGATACCTCA	
LA_Cr^10^ S1 forward (^10^S1_*f*_)	GAATAACTCGTTCGGAAAGCGT	
LA_Cr^10^ S4 reverse (^10^S4_*r*_)	GCAAAGAGAATTGTATTCCGTGT	
LA_Cr^11^ S1 forward (^11^S1_*f*_)	CACAACCGTGACAAATATTTGCA	
LA_Cr^11^ S2 reverse (^11^S2_*r*_)	CAATGTCGCGGATAAACTTAAGG	
LA_ Cr^12^ S1 forward (^12^S1_*f*_)	ACCCGGTTTGCATTTACCGAAG	
LA_Cr^12^ S2 reverse (^12^S2_*r*_)	ACAATCCCTCTAAATCTAGCCTCC	
LA3183 reverse	CTATCTAATGATTGGCCAACGC	Nested PCR (with ^7^S4_*r*_ or ^11^S1_*f*_ primer) to clone miniature CRISPR arrays (LA_Cr^6^ R2R4 or LA_Cr^12^ R2R3)
LA3189 upstream reverse	TCATTTTTCGGATTCCATTTTATT	
Repeat forward *Hin*dIII	CCCAAGCTTCTGAATATAACTTTGAT GCCGTTAGG	
Repeat reverse *Kpn*I	CGGGGTACCTTCTAAACCGCCTATCGGC	

### RNase assay with rLinCas6

The recombinant LinCas6 (rLinCas6) was purified using Ni-NTA (nitriloacetic acid) chromatography as described before (Prakash and Kumar, 2021). Cleavage assays with rLinCas6 ribonuclease were performed on the synthesized miniature pre-RNA substrates, as described previously (Prakash and Kumar, 2021). In brief, the synthesized miniature pre-crRNA (100 ng) was incubated with or without rLinCas6 (50-2000 nM) in a cleavage buffer (20 mM HEPES-KOH pH 8.0, 250 mM KCl, 1 mM DTT, and 2 mM MgCl_2_) for 1 h at 37°C. After that, cleavage reactions were terminated, resolved onto denaturing urea (8 M) 10% PAA gel, and were visualized with SYBR-Gold stain. Single-stranded RNA fragments of known sizes (24-500 nt) were used as a marker to estimate the size of the processed RNAs as described in a previous study (Prakash and Kumar, 2021).

## Results

### *In silico* analysis reveals seven CRISPR arrays at the subtype I-B locus of *L. interrogans* sv. Lai

**FIGURE 1:**
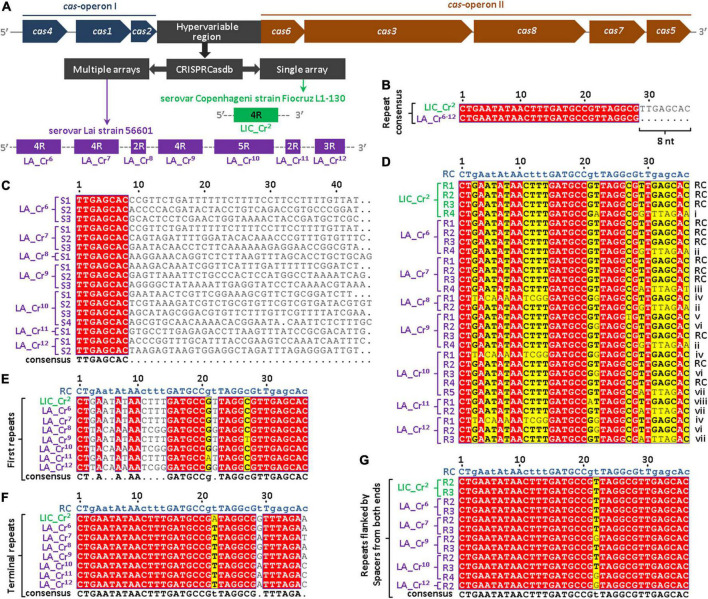
Multiple sequence alignments (MSA) of spacers and repeats of CRISPRs predicted in the I-B locus of *L. interrogans* serovar Lai strain 56601. **(A)** CRISPRCasdb database analysis of the hypervariable region in I-B locus of serovar Lai. The generalized architecture of the CRISPR-Cas I-B locus harbored by different strains of *L. interrogans* shows the hypervariable region that contains single or multiple CRISPRs, as observed in serovar Copenhageni (LIC_Cr^2^; Prakash and Kumar, 2021) or Lai (LA_Cr^6– 12^; in this study), respectively. Each CRISPR is represented by the number of associated repeats predicted by the CRISPRCasdb. **(B)** Nucleotide alignment of repeat consensus sequences of LIC_Cr^2^ and LA_Cr^6– 12^. Nucleotide alignment between database-defined repeat consensus of LIC_Cr^2^ and LA_Cr^6– 12^ showed a deficit of 8 nt at the 3′ end of LA_Cr^6– 12^ repeats. **(C)** MSA of spacer sequences. Spacer sequences (*n* = 17) of CRISPRs I-B (LA_Cr^6– 12^) in serovar Lai were retrieved from CRISPRCasdb and aligned using Clustal Omega. The alignment shows conserved 8 nt sequences at the 5′ end of each spacer (red filled box). **(D)** MSA of repeat sequences. Previously characterized and manually curated repeat sequences of CRISPRs I-B in serovar Copenhageni (LIC_Cr^2^) and Lai (LA_Cr^6– 12^), respectively, were aligned. The alignment shows conservation or polymorphism in the 1st to 36th nucleotide position of each repeat. Repeat consensus (RC) and repeat variants (i-viii) are indicated at the right to the respective sequences in the alignment. MSA of first repeats **(E)**, terminal repeats **(F)**, and repeats that are flanked by spacers at both ends **(G)**. Red and yellow colored nucleotides in the alignments represent 100% and more than 70% conservation, respectively, at that position among total sequences. Consensus sequences of each MSA were shown below the alignment where upper and lower cases denote conserved and semi-conserved nucleotides, respectively. Bold letter code in the alignments indicates consensus nucleotide. Dot in consensus sequences indicates no nucleotide conservation at that particular position.

In the genome of *L. interrogans*, the CRISPR subtype I-B locus is flanked by the two independent *cas*-operons (I and II) (Dixit et al., 2016) (Figure 1A). In this study, the intergenic region between *cas2* and *cas6* is called the hypervariable region that harbors the CRISPR I-B arrays of *L. interrogans*. Using the database CRISPRCasdb, a total of 11 CRISPR arrays (LIC_Cr^1–11^) were predicted in sv. Copenhageni. These CRISPR arrays provided by the CRISPRCasdb were numbered based on their serial order 1 to 11. Out of 11 CRISPR arrays (LIC_Cr^1–11^), a single CRISPR array (LIC_Cr^2^) (Prakash and Kumar, 2021) was predicted in the hypervariable region of *L. interrogans* sv. Copenhageni genome (Figure 1A and Supplementary Table 1). The remaining 10 arrays (LIC_Cr^1^ and LIC_Cr^3–11^) identified outside the hypervariable region were orphan CRISPR arrays. The array LIC_Cr^2^ comprises four repeats (36 nt each) interspaced by three unique spacers (Prakash and Kumar, 2021). On nucleotide BLAST analysis of LIC_Cr^2^ spacer sequences, no match was found in the other serovars of *L. interrogans*. This was consistent with a previous study (Xiao et al., 2019) where spacers were found variable across the serovars of *L. interrogans*. Moreover, a 100% nucleotides sequence identity was observed among the first three repeats, whereas the fourth repeat demonstrated variation at the 3′ end (Prakash and Kumar, 2021). Such variations persuaded us to analyze the repeats of CRISPR arrays in the hypervariable region of *L. interrogans* sv. Lai, another well-studied pathogenic serovar of *L. interrogans*. Unlike the genome of sv. Copenhageni, the sv. Lai harbored multiple arrays at the CRISPR-Cas I-B locus. Recently, in the CRISPR subtype I-B hypervariable region of sv. Lai, four CRISPR arrays with identical repeat consensus (28 nt) were predicted using the CRISPRFinder program (Xiao et al., 2019). In this study, an advanced version of the program CRISPRCasdb was used, which predicted 14 CRISPR arrays (LA_Cr^1–14^) in sv. Lai. Out of these 14 CRISPR arrays (LA_Cr^1–14^), 7 CRISPR arrays (LA_Cr^6–12^) were predicted in the hypervariable region of the sv. Lai genome (Figure 1A and Supplementary Table 1). The three extra arrays predicted in the hypervariable region of sv. Lai were LA_Cr^8^, Cr^11^, and Cr^12^. In sv. Lai, 7 (LA_Cr^1–5^ and LA_Cr^13–14^) out of the 14 arrays identified outside the hypervariable region were orphan CRISPR arrays. The CRISPRCasdb revealed that each repeat of the predicted CRISPR arrays (LA_Cr^6–12^) measured the size of 28 nucleotides. For any predicted CRISPR array, the CRISPRCasdb, by default, computes repeat consensus. Repeat consensus represents a sequence based on the occurrence of each nucleotide of repeats. The repeat consensus sequences provided by CRISPRCasdb of these arrays (LA_Cr^6–12^) were 100% identical (Supplementary Table 1). Interestingly, on alignment of the repeat consensus of the seven arrays, LA_Cr^6–12^ (28 nt) and LIC_Cr^2^ (36 nt), we noticed that at the 3′ end, each repeat of seven arrays (LA_Cr^6–12^) is deficit of eight nucleotides (Figure 1B). Compared to the 36 nt long repeats in array LIC_Cr^2^, shorter repeats (28 nt) in seven arrays LA_Cr^6–12^ incited to explore the spacer sequences of these seven arrays LA_Cr^6–12^. From the seven arrays LA_Cr^6–12^, a total of 17 spacer sequences were retrieved using the CRISPRCasdb. The retrieved 17 spacer sequences were used to perform MSA. In MSA, the 5′ ends of each of the 17 spacers were conserved by eight nucleotides (TTGAGCAC) (Figure 1C). This was in contrast to the observation where conserved repeats of the CRISPR array are separated by unique spacers (Mohamadi et al., 2020). Hence, to uphold the individuality in spacers and sequence conservation among repeats of seven arrays LA_Cr^6–12^, the conserved spacers sequence in this study has been redefined as part of adjacent repeats (Figure 1C). Such manual redefining of the spacers and repeats composition in the seven arrays (LA_Cr^6–12^) directed to increase repeats size (28 nt; program-defined) to 36 nucleotides. In addition, in order to maintain the consistency of repeat size (36 nt), the terminal repeats of each CRISPR array in the sv. Lai genome were extended at the 3′ end by eight nucleotides.

The direction of pre-crRNA transcription of LIC_Cr^2^ is not defined as per the CRISPRCasdb (Supplementary Table 1) (Prakash and Kumar, 2021). In a recent study, the direction of pre-crRNA transcription of LIC_Cr^2^ was demonstrated through RT-PCR and found to align with the direction of associated *cas* operon (Prakash and Kumar, 2021). We thus hypothesized that CRISPR arrays and *cas* genes of the subtype I-B system might also be co-directional among other *Leptospira* serovars or strains. However, CRISPRCasdb predicted the direction of pre-crRNA transcription for the seven arrays LA_Cr^6–12^ opposite to the *cas* genes (Supplementary Table 1). Thus, the CRISPRCasdb erred in projecting a defined and correct orientation of pre-crRNA in serovar Copenhageni (Prakash and Kumar, 2021) and Lai (in this study), respectively.

In this study, the previously characterized repeats (*n* = 4) of array LIC_Cr^2^ (Prakash and Kumar, 2021) and the repeats (*n* = 24) of seven arrays LA_Cr^6–12^ were manually curated and aligned to address the variation in the repeats. MSA of repeats (*n* = 4 + 24) from eight arrays (LIC_Cr^2^ and LA_Cr^6–12^) generated a repeat consensus (RC) of 36 nucleotides (Figure 1D). Analysis of the RC’s nucleotides demonstrated that each nucleotide is conserved by more than 70%. The nucleotides forming the stem-loop of the RC (Prakash and Kumar, 2021) were conserved in most of the repeat sequences (Figure 1D). Although 12 out of 28 repeats were identical to RC, variants of the repeat (RVs; *n* = 8) were also identified in sv. Copenhageni (i) and sv. Lai (ii-viii) at the hypervariable region (Figure 1D). Out of eight RVs (i-viii) identified, three RVs (v, vi, and viii) had a single nucleotide polymorphism. In contrast, the five RVs (i-iv and vii) showed variations of five to nine nucleotides, all coincidentally located either at first or terminal repeats of the arrays. Hence, a separate MSA of first and terminal repeats was conducted as shown in Figures 1E,F, respectively. The MSA with the RC demonstrated variation in nucleotides sequence primarily at 5′ of the first repeats (Figure 1E) and 3′ ends of terminal repeats (Figure 1F). In this study, thus the most conserved repeats of sv. Copenhageni and Lai genomes were the ones that possessed spacers at either end (Figure 1G).

### Comparative analysis of the spirochete genome at the hypervariable region

**FIGURE 2:**
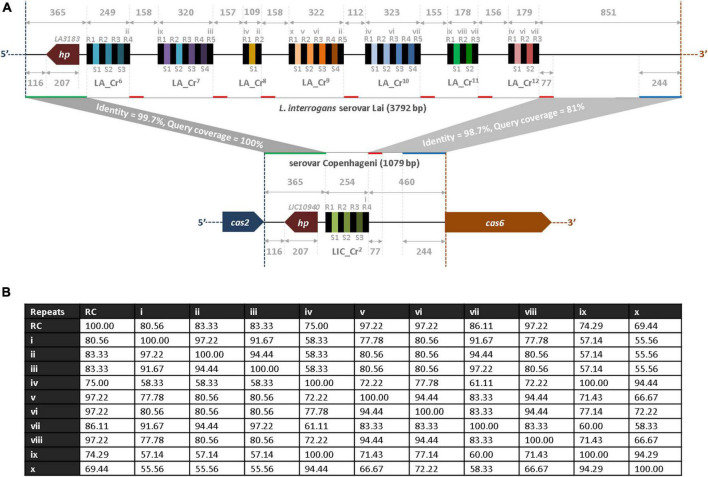
Bioinformatics analysis of hypervariable region at subtype I-B loci between *L. interrogans* sv. Lai and Copenhageni. **(A)** Comparison of the hypervariable region between *L. interrogans* serovars, Lai and Copenhageni. The region between *cas2* (*LA3182* and *LIC10941*) and *cas6* (*LA3189* and *LIC10939*) orthologs in serovar Lai (3,792 bp; top panel) and Copenhageni (1,079 bp; bottom panel) are drawn (manually) to scale in the 5′-3′ direction of the CRISPR-Cas I-B. Highly similar regions between serovar Lai and Copenhageni are shown by the same color-coded horizontal lines (green, red, and blue) along the lines (solid gray) that correspond to the hypervariable region in *Leptospira* serovars. Identical genes (*hp*; *LIC10940* and *LA3183*) in both strains are denoted by the dark red color-filled pentagon. Black and unique color-filled rectangles represent similar repeats and spacer regions in all CRISPRs. Different variants of repeats (i-x), except the repeats identical to the consensus sequence, are indicated over the black color-filled rectangles in the architecture. **(B)** Identity matrix of repeat consensus (RC) and variants. A total of 11 repeat sequences (RC and variants i-x) were aligned and an identity matrix was generated using the MAFFT program. The numbers in the matrix correspond to the percent identity between the two respective repeat sequences.

Identification of multiple CRISPR arrays at the hypervariable region of sv. Lai’s genome prompted us to perform a comparative analysis of the nucleotides sequences with the genome of sv. Copenhageni. The hypervariable sequences (3,792 bp) of the sv. Lai genome were obtained from the NCBI database, which encompasses the region between *cas2* (3′ end) and *cas6* (5′ end) of CRISPR-Cas subtype I-B (Supplementary Figure 1A). In the hypervariable region, the inter-array sequences range from 112 to 229 nucleotides, while each array is 108 to 323 nucleotides in size. The alignment of the five inter-array sequences (LA_Cr^6^-Cr^7^, LA_Cr^7^-Cr^8^, LA_Cr^8^-Cr^9^, LA_Cr^9^-Cr^10^, and LA_Cr^10^-Cr^11^) show similarity in the first 155 to 158 nucleotides from the 5′ end (Supplementary Figure 1B). However, beyond 155 to 158 nucleotides, three inter-arrays (LA_Cr^6^-Cr^7^, LA_Cr^8^-Cr^9^, and LA_Cr^10^-Cr^11^) demonstrated to possess additional repeat (∼94% identity) and the spacer-like sequences (36 nt). The repeat-like sequences were identical to one proposed RV (iv). Therefore, these newly identified repeat- and the spacer-like sequences (36 nt) were redefined as first-repeat and –spacers for the three (LA_Cr^7^, Cr^9^, and Cr^11^) arrays (Supplementary Figures 1B, 2A, and Supplementary Table 2). Such redefining of repeat and spacers resulted in the addition of two more RVs (ix and x) at the hypervariable region of sv. Copenhageni and Lai (Table 2). We also generated an identity matrix of RC and various RVs, where the RC sequence shared ∼69 to 97% identity with the list of RVs denoted as i-x (Figure 2B). However, the identity perceived among RVs ranges from ∼56 to 100%.

**TABLE 2:** CRISPR I-B repeats variants in *L. interrogans* svs. Copenhageni and Lai (in redefined CRISPR locus).

Repeat types	CRISPR repeats	Sequences (sense, 5′–3′)
RC (repeat consensus)	LIC_Cr^2^ R1, 2, and 3 LA_Cr^6^ R1, 2, and 3 LA_Cr^7^ R2, 3, and 4 LA_Cr^9^ R4 LA_Cr^10^ R2, and 4	CTGAATATAACTTTGATGCCGTTAGGCGTTGAGCAC
i	LIC_Cr^2^ R4	CTGAATATAACTTTGATGCCGATAGGCGGTTTAGAA
ii	LA_Cr^6^ R4 LA_Cr^8^ R2 LA_Cr^9^ R5	CTGAATATAACTTTGATGCCGTTAGGCGGTTTAGAA
iii	LA_Cr^7^ R5	CTGAATATAACTTTGATGCCGTTAGGCGATTTAGAT
iv	LA_Cr^8^ R1 LA_Cr^10^ R1 LA_Cr^12^ R1	CTTACAAAAATCGGGATGCCGGTAGGCGTTGAGCAC
v	LA_Cr^9^ R2	CTGAATATAACTTTGATGCCGTTAGGTGTTGAGCAC
vi	LA_Cr^9^ R3 LA_Cr^10^ R3 LA_Cr^12^ R2	CTGAATATAACTTTGATGCCGGTAGGCGTTGAGCAC
vii	LA_Cr^10^ R5 LA_Cr^11^ R3 LA_Cr^12^ R3	CTGAATATAACTTTGATGCCGTTAGGCGATTTAGAC
viii	LA_Cr^11^ R2	CTGAATATAACTTTGATGCCATTAGGCGTTGAGCAC
ix	LA_Cr^7^ R1 LA_Cr^11^ R1	CTTACAAAAATCGGG-TGCCGGTAGGCGTTGAGCAC
x	LA_Cr^9^ R1	CTTACAAAAATCGGGATGTCGGTAGGCGTTGAGTAC

Underlines and hyphens show polymorphism and deletion of nucleotide, respectively, in repeat variants compared to the repeat consensus.

At the hypervariable locus of sv. Lai and Copenhageni, the region between *cas2* and its proximal repeat (R1 of LA_Cr^6^ and LIC_Cr^2^) is 365 bp long and is highly conserved (99.7% sequence identity and 100% query coverage), as illustrated in Figure 2A. Whereas, the region between *cas6* and its proximal repeat (R3 of LA_Cr^12^ and R4 of LIC_Cr^2^) are 851 and 460 bp long with 98.7% sequence identity (81% query coverage). Within this region, around 244 bp upstream to *cas6* and 77 bp downstream to a proximal repeat of *cas6* are highly similar between serovar Lai and Copenhageni (Figure 2A). Interestingly, the DNA segment of 77 bp downstream to LIC_Cr^2^ aligned with high similarity to the downstream DNA segment of each seven arrays LA_Cr^6–12^ (Figure 2A). Such an identical feature in the hypervariable region suggests that the downstream DNA segment (77 bp, denoted by the red line) of each CRISPR array in serovar Lai and Copenhageni is conserved (Figure 2A).

### CRISPR arrays at the hypervariable region of serovar Lai are transcriptionally active

**FIGURE 3:**
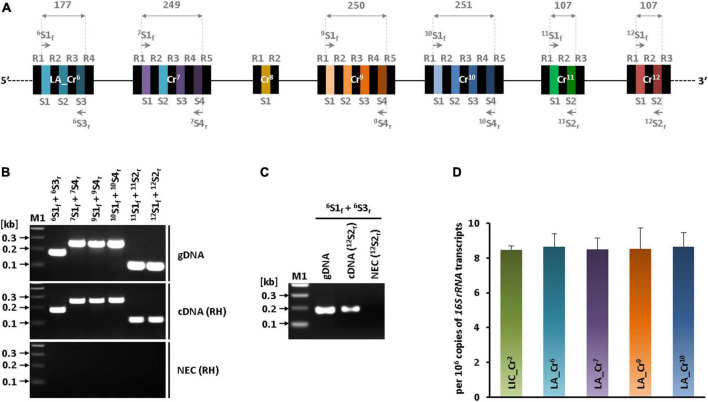
RT-PCR and qPCR of subtype I-B CRISPRs. **(A)** Schematic representation of primer pairs position used in the study. Spacer-specific primer pairs used in the RT-PCR experiment are denoted on the hypervariable region (I-B) of serovar Lai. CRISPR regions and length of fragments that were expected to get amplified with primers used in the study are indicated by vertical dashed lines (gray) and numbers (in bp) given at the apex of the double arrowhead over the architecture. **(B)** Identification of subtype I-B CRISPRs transcription in serovar Lai. PCR using genomic DNA (gDNA; positive control) of serovar Lai with the spacer (first and terminal) specific primer pair of each CRISPR (except LA_Cr^8^) (top panel). PCR with cDNA template synthesized from total RNA of serovar Lai using random hexamers (middle panel). PCR with RNA (cDNA synthesis reaction without reverse transcriptase) is a no enzyme control (NEC) of the experiment (bottom panel). PCR products were resolved on 2% agarose gel. “M1” denotes the DNA marker used for size estimation of PCR-amplified DNA fragments. **(C)** RT-PCR of first CRISPR using cDNA prepared using spacer-specific primer of terminal CRISPR of LA_Cr^6– 12^ series. A reverse transcriptase reaction was performed using a terminal spacer-specific reverse primer of LA_Cr^12^. This cDNA template was used in PCR for amplification of LA_Cr^6^. **(D)** Quantification of transcripts of CRISPR I-B arrays of sv. Copenhageni and Lai. Transcripts of CRISPR arrays (LIC_Cr^2^, LA_Cr^6^, Cr^7^, Cr^9^, and Cr^10^) are quantified using qRT-PCR per 10^6^ copies of *16 S rRNA* transcripts of respective *Leptospira* serovars. Results are indicative of two independent experiments, each performed in quadruplets.

The transcripts of seven CRISPR arrays (LA_Cr^6–12^) at the hypervariable region were determined by RT-PCR. The primer pairs used in RT-PCR were synthesized such that they anneal to the first and terminal spacers of each array (Table 1 and Figure 3A). The specificity of these primer sets was first tested by PCR using the genomic DNA of sv. Lai as a template. Expected DNA amplicons of 177 bp (LA_Cr^6^ S1S3), 249 bp (LA_Cr^7^ S1S4), 250 bp (LA_Cr^9^ S1S4), 251 bp (LA_Cr^10^ S1S4), and 107 bp (LA_Cr^11^ S1S2 and LA_Cr^12^ S1S2) were obtained using genomic DNA (Figures 3A,B, top panel). In agreement, similar sizes of amplicons were obtained from the two-step RT-PCR reaction, where cDNA was made using a random hexamer (Figure 3B, middle panel). No template amplification in another control RT-PCR reaction devoid of reverse transcriptase suggested RNA was free of DNA contamination (Figure 3B, bottom panel). Thus, we confirmed the active transcription of seven CRISPR arrays (LA_Cr^6–12^) at the hypervariable region of sv. Lai. However, due to long inter-array regions (112 to 158 nucleotides), it was unclear whether these seven CRISPR arrays (LA_Cr^6–12^) are transcribed as a single long pre-crRNA or as multiple independent pre-crRNA. Therefore, another set of PCR was performed to amplify consecutive arrays with a partial overlapping CRISPR region, as presented graphically in Supplementary Figure 2A. The partial overlapping region was amplified using the primer pairs enlisted in Supplementary Table 3 and the cDNA (random hexamer) as a template. The amplicons of size (655, 955, and 685 bp) could be detected for three overlapping CRISPR regions of LA_Cr^6^ S1-Cr^7^ S4, LA_Cr^7–9^, and LA_Cr^9–10^, respectively (Supplementary Figure 2B). We additionally substantiated our finding by generating another set of cDNA, where instead of random hexamer (RH), we used a single primer (^12^S2_*r*_) to the spacer region of the terminal array (LA_Cr^12^). After that, PCR was performed with the specific primer pair (^6^S1_*f*_ and ^6^S3_*r*_) of the first array (LA_Cr^6^) and the new set of cDNA (^12^S2_*r*_) as a template. A DNA amplicon of 177 bp was detected (Figure 3C), and thus, transcription of seven CRISPR arrays (LA_Cr^6–12^) in sv. Lai as a single long pre-RNA of CRISPR subtype I-B was ascertained from two independent approaches.

Next, the abundance of pre-crRNA of CRISPR subtype I-B in sv. Copenhageni and Lai were assessed using quantitative real-time PCR. The cDNA used was synthesized using random hexamers, and the primer pairs designed gave amplicon in the range of ∼180 to 250 bp size of subtype I-B arrays (LIC_Cr^2^ of sv. Copenhageni, LA_Cr^6^, Cr^7^, Cr^9^, and Cr^10^ of sv. Lai). The quantified number of pre-crRNA in both serovars was more than 8 per 10^6^ copies of *16S rRNA* of *L. interrogans* (Figure 3D). The relative number of precursor RNA transcripts (CRISPR subtype I-B) in serovar Lai substantiates the RT-PCR analysis results in this study that demonstrated transcription CRISPR cluster jointly as a single precursor unit.

### Recombinant LinCas6 of serovar Copenhageni processes the pre-crRNA transcripts of serovar Lai

**FIGURE 4:**
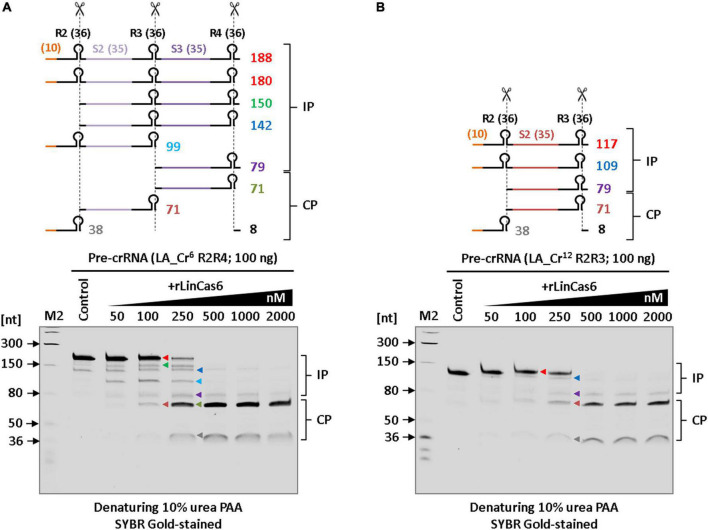
*In vitro* processing of the miniature CRISPR I-B transcript of serovar Lai with rLinCas6 endoribonuclease. **(A)** Nuclease activity of rLinCas6 on pre-crRNA of LA_Cr^6^. *In vitro* synthesized LA_Cr^6^ miniature transcript (R2R4; 188 nt) was incubated with increasing concentrations of rLinCas6 (50–2,000 nM). The possible pre-crRNA derived cleaved fragments (*n* = 9) by rLinCas6 are illustrated as incompletely processed (IP; *n* = 5) and completely processed (CP; *n* = 4) fragments (top panel). The reaction products were analyzed on denaturing urea gel after staining with SYBR Gold. At 100 to 250 nM of rLinCas6, six bands of different molecular lengths, each marked with unique color, were detected (bottom panel). **(B)** Nuclease activity of rLinCas6 on miniature pre-crRNA of LA_Cr^12^ (R3R4). IP and CP fragments on the gel were mapped and indicated right to the gel images. Repeats and spacers are shown by black and unique colors, respectively, in the pre-crRNA outline. The orange line denotes a vector-derived additional 10 nt at its 5′ end of each pre-crRNA. All reactions, including controls (no protein), were incubated for 1 h at 37°C. “M2” denotes RNA markers used for the size estimation of RNA fragments observed on the gels.

Using the endoribonuclease activity of rLinCas6, mature crRNAs can be generated from pre-crRNA (LIC_Cr^2^) of sv. Copenhageni, as described previously (Prakash and Kumar, 2021). In LinCas6 (LIC10939), potential catalytic residues and glycine rich-loop (G-loop) are crucial for self-folding and RNA substrate recognition (Prakash and Kumar, 2021). LinCas6 in sv. Copenhageni (LIC10939) and Lai (LA3189) share 96.2% amino acid sequence identity with 100% query coverage (210 residues). Pairwise alignment of LIC10939 and LA3189 revealed a mismatch at eight residues (T36, Q65, T124, I143, Q146, K149, V184, and S202) in LA3189. These mismatches in LA3189 were not observed at the potential catalytic triad and G-loop. Therefore, to address whether the CRISPR arrays in sv. Lai can be processed to yield mature crRNAs; we opted to use rLinCas6 (LIC10939) endonuclease. Previously, a miniature form of pre-crRNA has been successfully used in the RNase assay of Cas6 protein (Reimann et al., 2017). Similarly, an *in vitro* cleavage assay was set up in which miniature pre-crRNA of the LA_Cr^6^ array (R2R4) was incubated with increasing concentrations of rLinCas6. For clarity, the total feasible RNA fragments (n = 9) after processing the miniature pre-crRNA (188 nt) of LA_Cr^6^ (R2R4) with rLinCas6 were mapped for cleavage reaction analysis (Figure 4A, top panel). These transcript fragments have been categorized as incompletely processed (IP; *n* = 5, 79-180 nt) and completely processed (CP; *n* = 4, 8-71 nt) fragments, as described previously (Prakash and Kumar, 2021). The miniature pre-crRNA cleavage by rLinCas6 on denaturing urea-PAGE revealed six distinct bands (Figure. 4A, bottom panel). Larger IP fragments of pre-crRNA were identified when rLinCas6 was employed at a lower range of concentrations (50–250 nM), whereas at a higher concentration of rLinCas6 (500-2000 nM), an increase in the intensity of CP fragments of partial pre-crRNA was observed. RNA fragments of 71 nucleotides in CP products illustrate the generation of mature crRNAs from the miniature LA_Cr^6^ transcript. Similarly, rLinCas6-mediated mature crRNA biogenesis was also investigated on the miniature LA_Cr^12^ (R2R3) array (Figure 4B). Alike the miniature LA_Cr^6^ processing, RNA fragments of 71 nucleotides in CP products could be detected for the miniature LA_Cr^12^ (R2R3) array. Thus, with the processing results of the miniature versions of LA_Cr^6^ and LA_Cr^12^, we infer that rLinCas6 may process the remaining five array transcripts of serovar Lai to yield mature crRNAs.

### *In silico* analysis of spacers of I-B array identified a consensus PAM

**FIGURE 5:**
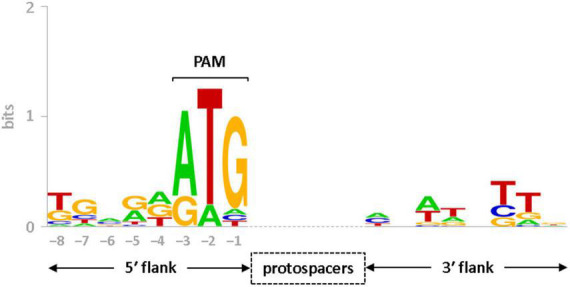
Sequence logo of protospacer flanks. Conservation of nucleotides at the 5′ and 3′ flanks (8 nt each) of protospacers, corresponding to the spacers of seven CRISPR arrays (LA_Cr^6– 12^) in serovar Lai, are presented in the form of sequence logo. Conserved nucleotides at –3 (A), –2 (T), and –1 (G) positions in the upstream of protospacers represent consensus PAM sequence (5′-ATG-3′). The vertical axis indicates the information content of the sequence position in bits. The height of each letter in the logo represents the conservation of that nucleotide at each position.

CRISPR-Cas systems rely on protospacer adjacent motif (PAM) to differentiate between self (spacer) and non-self (protospacer) DNA sequences (Westra et al., 2013). In CRISPR-Cas type I systems, PAMs are often reported to be present at the 5′-end of the protospacers sequence (Deveau et al., 2008; Mojica et al., 2009). In this study, the spacer sequences and CRISPR boundaries of sv. Lai’s genome has been redefined manually. Therefore, the rectified spacer sequences (Supplementary Table 2) were utilized to identify PAMs for the subtype I-B system of *Leptospira* by *in silico* approach. Using the CRISPRTarget program, analysis of rectified spacers of the I-B array obtained 53 hits as possible protospacers (with a cut-off score of 25). These hits aligned with viral genome fragments derived from metagenomic samples and *Leptospira* phages (LinZ_10, Lin_34, LbrZ_5399, LnoZ_CZ214). Further, by the upstream of a majority of predicted protospacers (37 out of 53; 70% of hits), a trinucleotide ATG was conserved, as evident in the sequence logo of nucleotides flanking the protospacers (Figure 5). Therefore, we speculate that one of the PAM (5′-ATG-3′) may be employed for the interference study against mobile genetic elements in *Leptospira*.

## Discussion

Understanding the CRISPR transcript orientation and the repeat-spacer junctions is valuable for the evolving genome editing techniques, selective killing, and gene expression modulation (Zheng et al., 2020). Two of the most accustomed programs for searching CRISPR arrays or Cas loci in prokaryotic genomes are the CRISPRFinder (Grissa et al., 2007) and CRISPRCasFinder (Pourcel et al., 2020). Using the CRISPRFinder program, recently, four CRISPR arrays (LA_Cr^6^, Cr^7^, Cr^9^, and Cr^10^) have been reported in the hypervariable region of *L. interrogans* sv. Lai genome (Xiao et al., 2019). On the other hand, in this study, using the more robust CRISPRCasdb program, three more CRISPR arrays (LA_Cr^8^, Cr^11^, and Cr^12^) were predicted. Although the CRISPRCasdb program accurately predicted the repeat-spacer junctions of the CRISPR subtype I-B array in the sv. Copenhageni, it failed to provide the correct repeat-spacer junctions or the orientation of arrays in sv. Lai genome.

Integrating protospacer DNA into the CRISPR array is an essential and primary stage of the CRISPR-Cas-mediated adaptive immunity for developing memory against mobile genetic elements (Mosterd et al., 2021). Such integration depends on the length of leader elements driving the array or the consensus within repeat nucleotides. The length of the leader sequences is reported from 100 to 500 nucleotides (Yosef et al., 2012; Carte et al., 2014); however, 10 to 43 nucleotides of the leader at the leader-repeat junction are critical for adaptation (Yosef et al., 2012; Wei et al., 2015). Also, mutation of the eight nucleotides of the repeat at the leader junction disrupts the adaptation process (Grainy et al., 2019). In sv. Lai, identifying transcripts from seven CRISPR arrays (LA_Cr^6^ to Cr^12^) by RT-PCR suggested that a single leader controls these arrays. In agreement, the nucleotide sequences flanking the 5′ end of the first array (LA_Cr^6^), where a leader is expected, differ from the remaining six CRISPR arrays (LA_Cr^7–12^) present in the hypervariable region.

In a study done elsewhere (Barrangou et al., 2007), it is observed that repeats are often highly conserved within a given CRISPR array except for the terminal repeat. In contrast, multiple (n = 9) repeat variants (RV) were found in the CRISPR arrays of sv. Lai. The first repeat of array LA_Cr^6^ resembles the RC; however, in the remaining six CRISPR arrays (LA_Cr^7–12^), the first repeats differed from the RC at the 5′ end. Therefore, such variations at the first repeats of six CRISPR arrays (LA_Cr^7–12^) in sv. Lai may disrupt the adaptation process, as reported elsewhere (Grainy et al., 2019). Based on earlier reported work (Grainy et al., 2019), a hypothesis thus can be drawn that during a new spacer integration in CRISPR-Cas subtype I-B of serovar Lai, adaptation may exclusively occur at the first CRISPR array (LA_Cr^6^). The biogenesis of crRNA from pre-crRNA (miniature LA_Cr^6^ and Cr^12^) and the conservation of the stem-loop of repeat RNA indicate that the interference process from all seven arrays (LA_Cr^6–12^) may still be possible in *Leptospira*. Such adaptation and interference study, however, needs to be experimentally proven and can be an exciting subject for future study.

The biogenesis of crRNAs in a CRISPR-Cas type I and its subtypes from pre-crRNA requires Cas6 endoribonucleases except for the subtype I-C where processing is done by Cas5 (Charpentier et al., 2015; Hochstrasser and Doudna, 2015). After processing, crRNA is loaded into a multi-protein effector complex, forming a “Cascade” that guides crRNA to the target (He et al., 2020). Cascade recruits Cas3 (signature protein of Type I) to form the “Interference” complex at the target and degrades the specific DNA (He et al., 2020). In general, Cas3 protein possess an N-terminal histidine-aspartate (HD) nuclease domain and a C-terminal superfamily 2 (SF2) helicase domain (Jackson et al., 2014; He et al., 2020). The SF2 domain contains a DEAD/DEAH box region. Multiple proteins having the DEAD-box family (SF2) of RNA helicases have been shown to resolve RNA secondary structures and unfold RNA hairpins *in vitro* (Rogers et al., 1999; Marsden et al., 2006; König et al., 2013). Interestingly, in *L. interrogans* sv. Linhai, Cas3 has been reported naturally fused with Cas6 (Prakash and Kumar, 2021). Therefore, Cas3 may also have some role during the expression stage, if not for all, at least in *Leptospira*. The existence of single long pre-crRNA arrays in sv. of *L. interrogans* may have some evolutionary role for the fusion stage of LinCas3 and LinCas6. It is likely that the long pre-crRNA may form a stable and compact structure as reported elsewhere (König et al., 2013) and thus may not be uniformly accessible to LinCas6. Thus, it is plausible that the processing of lengthy pre-crRNA may require another helper protein(s) to maintain a susceptible structure of pre-crRNA for LinCas6. We speculate that the helicase activity of LinCas3 may unwind the long pre-crRNA so that repeat RNA segments are accessible to LinCas6. However, further work is warranted to validate this hypothesis.

In addition to the eight CRISPR arrays (LIC_Cr^6^ and LA_Cr^6–12^) identified in the I-B loci of svs. Copenhageni and Lai, 17 CRISPR arrays were qualified as orphan arrays. It has been suggested that orphan CRISPR arrays can be non-functional or associated with a remotely located *cas* locus in the same genome (Zhang and Ye, 2017). However, the biological relevance of these orphan CRISPR arrays in *L. interrogans* remains to be elucidated via transcriptional analysis and processing by host Cas endoribonucleases.

In sv. Lai, using the redefined spacer sequences, we identified a conserved PAM sequence (5′-ATG-3′) for CRISPR-Cas subtype I-B. Another weakly conserved PAM sequence (5′-TAC-3′) was identified through the same approach in the *L. interrogans* subtype I-B elsewhere (Xiao et al., 2019). Such inconsistency in the prediction of consensus PAM could be due to feeding different spacer lengths or its orientation during *in silico* analysis. Recently, a computational pipeline (Vink et al., 2021) has predicted the functional orientation of spacer and its associated PAM (5′-ATG-3′) for the subtype I-B system of *Leptospira*, which agrees well with our results presented in this study and elsewhere (Prakash and Kumar, 2021).

Understanding the endogenous CRISPR-Cas system may be advantageous in developing a genetic tool to understand gene function in pathogenic *Leptospira* like other infectious organisms described before (Li et al., 2016; Pyne et al., 2016; Cheng et al., 2017; Maikova et al., 2019). In this study, we deciphered the transcription of CRISPR RNA in one of the reference serovars of *L. interrogans* (sv. Lai strain 56601) harboring CRISPR-Cas subtype I-B in its genome. Although various robust *in silico* CRISPR prediction tools have been developed to project the CRISPR direction and boundaries, we failed to apply the same in *L. interrogans* sv. Lai and other serovars. Using RT-PCR, we present the transcriptional analysis of a cluster of CRISPR arrays at the I-B locus in *L. interrogans* sv. Lai. Our study suggests that these CRISPR arrays are transcriptionally active and controlled by a single leader. We also demonstrated the generation of mature crRNAs from selective pre-crRNA suggesting crRNA biogenesis associated with the CRISPR-Cas I-B in sv. Lai. In addition, a conserved PAM sequence was predicted using the spacer sequences of sv. Lai. This study comprehends the knowledge of CRISPR locus transcription and its processing of spirochetes and will be valuable in the future study of the endogenous CRISPR-Cas system in *Leptospira*.

## Data availability statement

The original contributions presented in this study are included in the article/Supplementary material, further inquiries can be directed to the corresponding author.

## Author contributions

AP performed the research work. MK and AP wrote the manuscript. Both authors contributed to the article and approved the submitted version.
