# Supplementary material for: Transcriptional analysis of CRISPR I-B arrays of Leptospira interrogans serovar Lai and its processing by Cas6
Source: Front Microbiol. 2022 Jul 29;13:960559. doi: 10.3389/fmicb.2022.960559 (PMC9372919; doi:10.3389/fmicb.2022.960559)
Supplement: Supplementary file 1 [file Data_Sheet_1.PDF]

SUPPLEMENTARY INFORMATION

Transcriptional analysis of CRISPR I-B arrays of *Leptospira interrogans* serovar Lai and its processing by Cas6

Aman Prakash and Manish Kumar#

Department of Biosciences and Bioengineering, Indian Institute of Technology Guwahati,

Guwahati-781039, Assam, India

#corresponding author: Manish Kumar

Department of Biosciences and Bioengineering, Indian Institute of Technology Guwahati, Guwahati-781039, Assam, India

Email: mkumar1@iitg.ac.in

Phone: +91-361-258-2230

Fax: +91-361-258-2249

METHODS

Bioinformatics analysis

Information of predicted CRISPRs **(Table S1)** in the I-B locus of *L. interrogans* sv. Copenhageni and sv. Lai was retrieved via a taxonomic-based search in the CRISPRCasdb database, a repository built with CRISPRCasFinder [1]. Nucleotide sequences of interCRISPR regions at the hypervariable region of sv. Lai's genome was extracted from the NCBI nucleotide database.

Multiple sequence alignments of interCRISPR regions were performed using the MUSCLE program [2], and graphic images of aligned sequences were obtained using the ESPript program (version 3.0) [3]. Sequences of repeat-spacer segments retrieved from the redefined CRISPR arrays of sv. Lai (LA_Cr$^{6-12}$) are tabulated in **Table S2**.

RT-PCR

PCR was performed using a cDNA or negative control (RNA) template prepared in reverse transcription (random hexamers) with or without reverse transcriptase, respectively. Primer pairs selected to use in PCR were specific to spacer regions, as mentioned in **Table S3**. PCR products were resolved on 2% agarose gel.

Table S1. CRISPRs predicted by the CRISPRCasdb in the I-B locus of *L. interrogans* serovars Copenhageni (strain Fiocruz L1-130) and Lai (strain 56601)

L. interrogans **serovar (strain)**	**CRISPR (I-B) Id**	**Coordinate (start-end)**	**Spacer**	**Repeat consensus**	**CRISPR (I-B) Direction**	**Evidence level**	***cas*** **(I-B) ORFs Direction**
Copenhageni (Fiocruz L1-130)	AE016823.1_2 (LIC_Cr[2])	1,133,848-1,134,101	3	GTGCTCAACGCCTAACGGCATCAAAGTTATATTCAG	ND	4	−
Lai (56601)	AE010300.2_6 (LA_Cr[6])	3,163,254-3,163,495	3	CTGAATATAACTTTGATGCCGTTAGGCG	−	4	+
	AE010300.2_7 (LA_Cr[7])	3,163,731-3,163,973	3	CTGAATATAACTTTGATGCCGTTAGGCG	−	4	
	AE010300.2_8 (LA_Cr[8])	3,164,138-3,164,239	1	CTGAATATAACTTTGATGCCGTTAGGCG	−	4	
	AE010300.2_9 (LA_Cr[9])	3,164,476-3,164,719	3	CTGAATATAACTTTGATGCCGTTAGGCG	−	4	
	AE010300.2_10 (LA_Cr[10])	3,164,839-3,165,154	4	CTGAATATAACTTTGATGCCGTTAGGCG	−	3	
	AE010300.2_11 (LA_Cr[11])	3,165,387-3,165,487	1	CTGAATATAACTTTGATGCCGTTAGGCG	−	4	
	AE010300.2_12 (LA_Cr[12])	3,165,651-3,165,821	2	CTGAATATAACTTTGATGCCGTTAGGCG	−	4	

ND: not defined

Table S2. Repeats and spacers of redefined CRISPR I-B arrays at the hypervariable region of serovar Lai

CRISPRs	Repeats (5′-3′)	Spacers (5′-3′)
LA_Cr[6]	R1: CTGAATATAACTTTGATGCCGTTAGGCGTTGAGCAC<br>R2: CTGAATATAACTTTGATGCCGTTAGGCGTTGAGCAC<br>R3: CTGAATATAACTTTGATGCCGTTAGGCGTTGAGCAC<br>R4: CTGAATATAACTTTGATGCCGTTAGGCGGTTTAGAA	S1: CCGTTCTGATTTTTTCTTTTCCTTCCTTTTGTTAT<br>S2: ACCCCACGATACTACCTGTCAGACCGTGCCCGGAT<br>S3: GCACTCCTCGAACTGGTAAAACTACCGATGCTCGC
LA_Cr[7]	R1: CTTACAAAAATCGGGTGCCGGTAGGCGTTGAGCAC<br>R2: CTGAATATAACTTTGATGCCGTTAGGCGTTGAGCAC<br>R3: CTGAATATAACTTTGATGCCGTTAGGCGTTGAGCAC<br>R4: CTGAATATAACTTTGATGCCGTTAGGCGTTGAGCAC<br>R5: CTGAATATAACTTTGATGCCGTTAGGCGATTTAGAT	S1: TTGATTGGTGCAGTTGTGCTTGTTTTGATTGGTGT<br>S2: CCGTTCTGATTTTTTCTTTTCCTTCCTTTTGTTAT<br>S3: CAGTAGATTTTGGATACACAAACCCGTTTGTGTTTC<br>S4: GAATACAACCTCTTCAAAAAAGAGGAACCGGCGTA
LA_Cr[8]	R1: CTTACAAAAATCGGGATGCCGGTAGGCGTTGAGCAC<br>R2: CTGAATATAACTTTGATGCCGTTAGGCGGTTTAGAA	S1: AAGGAAACAGGTCTCTTAAGTTTAGCACCTGCTGCAG
LA_Cr[9]	R1: CTTACAAAAATCGGGATGTCGGTAGGCGTTGAGTAC<br>R2: CTGAATATAACTTTGATGCCGTTAGGTGTTGAGCAC<br>R3: CTGAATATAACTTTGATGCCGGTAGGCGTTGAGCAC<br>R4: CTGAATATAACTTTGATGCCGTTAGGCGTTGAGCAC<br>R5: CTGAATATAACTTTGATGCCGTTAGGCGGTTTAGAA	S1: TGAGTATGCAAATGAGCTTCGGCTTCGAATTCCCT<br>S2: AAAGACAAATCGGTTCATTTGATTTTTTCGGATCT<br>S3: GAGTTAAATTCTGCCCACTCCATGGCCTAAAATCAG<br>S4: AGGGGCTATAAAATTGAGGTATCCTCAAAACGTAAA
LA_Cr[10]	R1: CTTACAAAAATCGGGATGCCGGTAGGCGTTGAGCAC<br>R2: CTGAATATAACTTTGATGCCGTTAGGCGTTGAGCAC<br>R3: CTGAATATAACTTTGATGCCGGTAGGCGTTGAGCAC<br>R4: CTGAATATAACTTTGATGCCGTTAGGCGTTGAGCAC<br>R5: CTGAATATAACTTTGATGCCGTTAGGCGATTTAGAC	S1: GAATAACTCGTTCGGAAAGCGTTCTGCGGATCTT<br>S2: TCGTAAAGATCGTCTGCGTGTTCGTCGTGATACGTGT<br>S3: AGCATAGCGGACGTGTTCTTTGTTCGTTTTATCGAA<br>S4: AGCGTGCAACAAAACACGGAATACAATTCTCTTTGC
LA_Cr[11]	R1: CTTACAAAAATCGGGTGCCGGTAGGCGTTGAGCAC<br>R2: CTGAATATAACTTTGATGCCATTAGGCGTTGAGCAC<br>R3: CTGAATATAACTTTGATGCCGTTAGGCGATTTAGAC	S1: CACAACCGTGACAAATATTTGCAAATCGTTCGACT<br>S2: GTGCCTTGAGAGACCTTAAGTTTATCCGCGACATTG
LA_Cr[12]	R1: CTTACAAAAATCGGGATGCCGGTAGGCGTTGAGCAC<br>R2: CTGAATATAACTTTGATGCCGGTAGGCGTTGAGCAC<br>R3: CTGAATATAACTTTGATGCCGTTAGGCGATTTAGAC	S1: ACCCGGTTTGCATTTACCGAAGTCCAAATCAATTTC<br>S2: TAAGAGTAAGTGGAGGCTAGATTTAGAGGGATTGT

Table S3. Primer pairs used in the RT-PCR experiment

Primer pair	Sequence (5′-3′)	Region amplified
LA_Cr^{6} S1 forward ($^{6}S1_{f}$)<br>LA_Cr^{7} S4 reverse ($^{7}S4_{r}$)	CCGTTCTGATTTTTTCTTTTCCT<br>TACGCCGGTTCCTCTTTTTTG	LA_Cr^{6} S1 to Cr^{7} S4
LA_Cr^{7} S2 forward ($^{7}S2_{f}$)<br>LA_Cr^{9} S4 reverse ($^{9}S4_{r}$)	CCGTTCTGATTTTTTCTTTTCCT<br>TTTACGTTTTGAGGATACCTCA	LA_Cr^{7} S2 to Cr^{9} S4
LA_Cr^{9} S2 forward ($^{9}S2_{f}$)<br>LA_Cr^{10} S4 reverse ($^{10}S4_{r}$)	AAAGACAAATCGGTTCATTTGA<br>TTTACGTTTTGAGGATACCTCA	LA_Cr^{9} S2 to Cr^{10} S4

REFERENCES

[1] C. Pourcel, M. Touchon, N. Villeriot, J.-P. Vernadet, D. Couvin, C. Toffano-Nioche, G. Vergnaud, Nucleic acids research, 48 (2020) D535-D544.
[2] F. Madeira, Y.M. Park, J. Lee, N. Buso, T. Gur, N. Madhusoodanan, P. Basutkar, A.R.N. Tivey, S.C. Potter, R.D. Finn, R. Lopez, Nucleic acids research, 47 (2019) W636-W641.
[3] X. Robert, P. Gouet, Nucleic acids research, 42 (2014) W320-W324.

LEGENDS TO SUPPLEMENTARY FIGURES

Fig. S1. Analysis of interCRISPR regions at the hypervariable region of serovar Lai. (A) The architecture of subtype I-B array locus in serovar Lai. CRISPRs identified in hypervariable (the region between *cas2*/*LA3182* and cas6/*LA3189*) of serovar Lai via the CRISPRCasdb database are drawn in the direction of CRISPR-Cas (5′-3′). CRISPRs and *cas* genes are shown in the architecture to the scale, except for flanking regions. Black and unique color-filled rectangles represent similar repeats and spacer regions, respectively. Length of CRISPR and flanking regions are indicated by vertical dashed lines (grey) and numbers (in bp) given at the apex of the double arrowhead over the architecture. (B) Multiple sequence alignment (MSA) of regions flanked by two adjacent CRISPRs. Nucleotide sequences flanked by LA_Cr$^{6\text{ and }7}$, LA_Cr$^{7\text{ and }8}$, LA_Cr$^{8\text{ and }9}$, LA_Cr$^{9\text{ and }10}$, and LA_Cr$^{11\text{ and }12}$ were aligned. Grey and unique colored regions at the 3′ terminus of alignment represent the first repeat-spacer units (redefined now) of LA_Cr^{7}, Cr^{9}, and Cr^{11}. Red and yellow colored nucleotides in the alignments represent 100% and more than 70% conservation, respectively, at that position among whole sequences. Consensus sequences of each MSA were shown below the alignment where upper and lower case denotes conserved and semi-conserved nucleotides, respectively. Bold letter code in the alignments indicates consensus nucleotide. Dot in consensus sequences indicates no nucleotide conservation at that particular position.

Fig. S2. RT-PCR of CRISPR I-B of serovar Lai. (A) Schematic representation of primer's position used to amplify cDNA of serovar Lai. The primers used in RT-PCR analysis and corresponding annealing regions are indicated over the graphic for clarity. LA_Cr^{6} to LA_Cr^{12}, including CRISPR flanking regions, are drawn to scale in the direction of CRISPR-Cas I-B (5′-3′). Similar repeats and distinct spacers in CRISPRs are represented by black and unique color-filled rectangles, respectively. Vertical dashed lines (grey) and numbers (in bp) given at the apex of the double arrowhead over the architecture indicate DNA regions and corresponding lengths that could be amplified in RT-PCR. (B) Identification of transcription from one CRISPR to another in serovar Lai. The primer pairs aforementioned were used in PCR with cDNA (right panel) or a control template (right panel) prepared using random hexamers. PCR products were resolved on 2% agarose gel. 'M1' denotes the DNA marker used for the size estimation of PCR-amplified fragments.
