# Supplementary figures and images for: Transcriptional analysis of CRISPR I-B arrays of Leptospira interrogans serovar Lai and its processing by Cas6
Source: Front Microbiol. 2022 Jul 29;13:960559. doi: 10.3389/fmicb.2022.960559 (PMC9372919; doi:10.3389/fmicb.2022.960559)

**Figure S1**

**(A)**

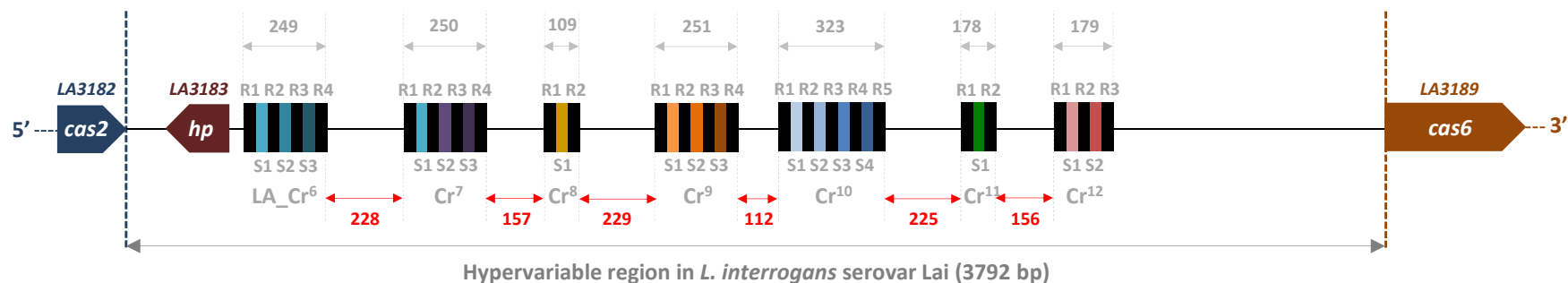

**(B)**

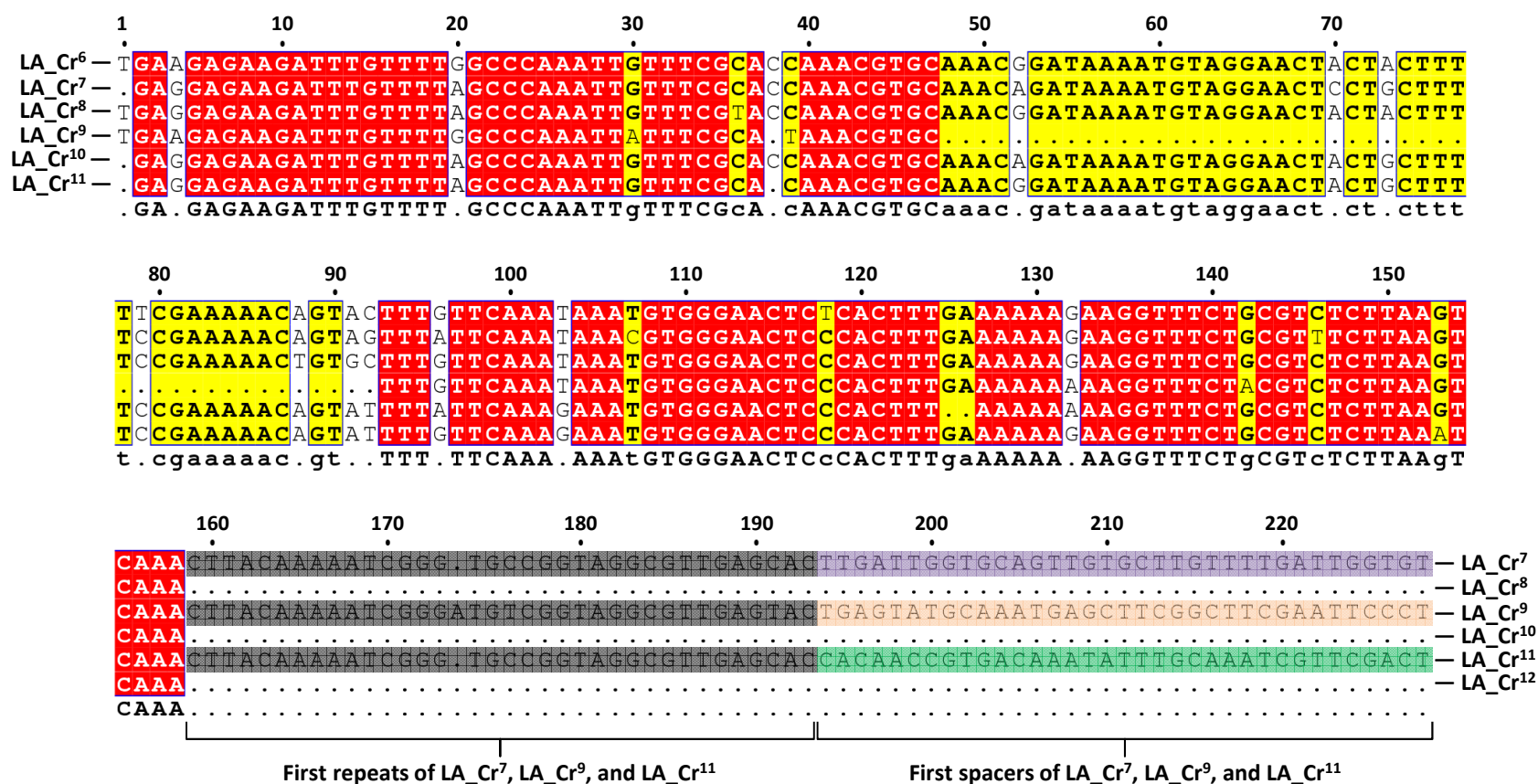

Figure S2

(A)

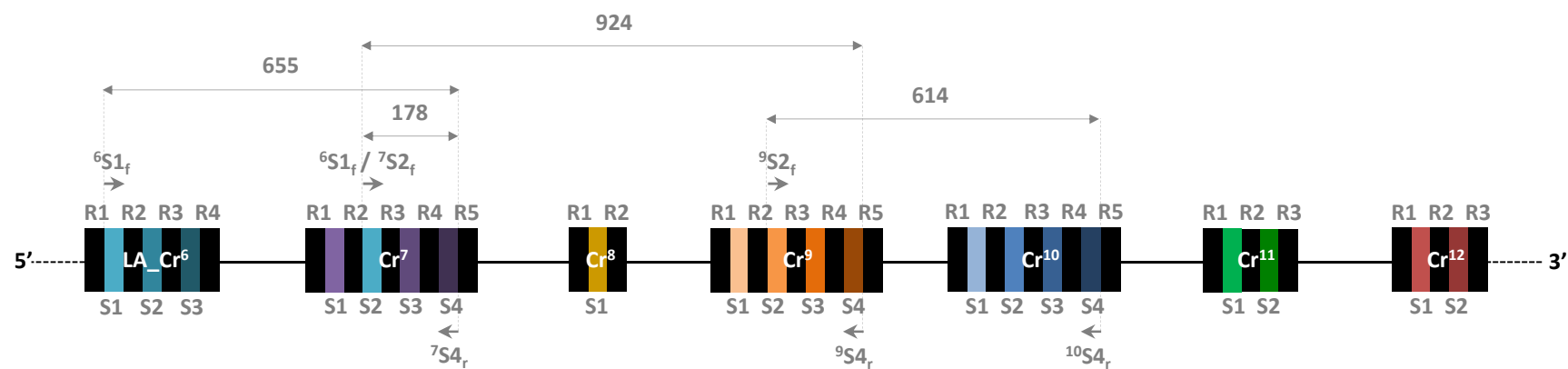

(B)

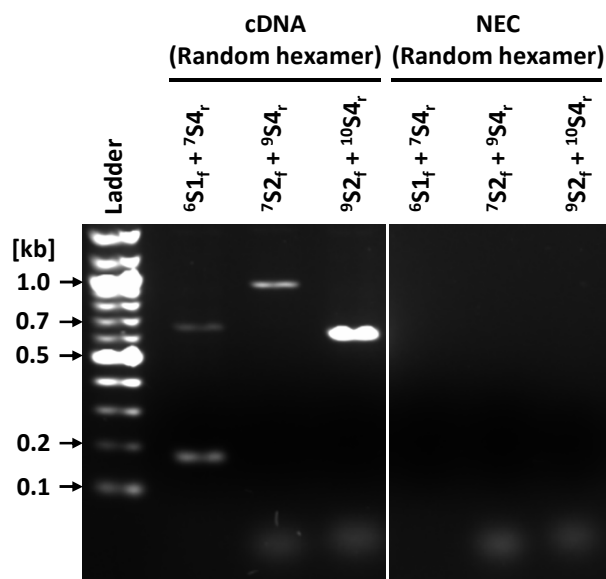

Supplement: Supplementary file 2 [file Data_Sheet_2.PDF]
